# Addressing the humanitarian crisis in Afghanistan through $10 billion Afghani assets: what are the challenges and opportunities at hand?

**DOI:** 10.1186/s12992-022-00868-8

**Published:** 2022-07-30

**Authors:** Mohammad Yasir Essar, Henry Ashworth, Arash Nemat

**Affiliations:** 1 Kabul University of Medical Sciences, Kabul, Afghanistan; 2 Alameda Health System, Highland Hospital, Department of Emergency Medicine, Oakland, California USA

**Keywords:** Afghanistan, Humanitarian crisis, Economic crises, Globalization, Healthcare, Education

## Abstract

**Background:**

The current humanitarian crisis in Afghanistan started after the US and international allies’ withdrawal. This has put the country in a dire situation as the globalized infrastructure supporting Afghanistan came to halt. Moreover, 10 billion USD of Afghanistan’s assets were frozen by the U.S and other international organizations after the Taliban takeover. This further exacerbated the humanitarian crisis and quickened the economic collapse in Afghanistan. These assets should be freed to support the people of Afghanistan.

**Main body:**

In order to address this situation, international oversight is needed to allow these funds to be returned and used by the Central Bank of Afghanistan without misappropriation by the Taliban. We suggest a number of short term interventions and long term considerations to improve the situation in Afghanistan with the $10 billion in frozen assets. In the short term, economic stability and the hunger crisis should be addressed by funding international organizations such as the World Food Program and national Afghani NGOs. In the long term funds should be used to build back the economy, build healthcare infrastructure, and support the development of women and children.

**Conclusion:**

At this juncture, the world and international organizations have a moral and ethical responsibility to ensure the 10 billion in funds go to the owners, the people of Afghanistan. With oversight and fund distribution to the right partners, progress can be made by providing support in security, healthcare, education and food resources. This calls for action to deliver $10 billion of assets to the Afghan people in a transparent manner, avoiding further tension and disasters in the country.

## Introduction

Humanitarian crises and their mitigation are becoming more inextricably tied to globalization and geopolitics. As nation states often cause crises through war or persecution of citizens, we rely on international organizations and NGOs to address the outcomes of these crises [1]. This cycle does not address the underlying cause of crises, but further perpetuates the need for a global response. This cycle is no more evident than in Afghanistan. It is important for us to learn lessons from the impacts globalization has had on the economy and health of Afghanistan in order to prevent further disasters in the future.

After the US withdraw a total 10 of billion US dollars in assets, once held overseas by the Central Bank of Afghanistan, has now been frozen [2] as the US and other international allies understandably do not want these assets to fall into the hands of the Taliban. This presents a global ethical problem on who has a right to the assets and what should be done with them. Already the US has divided these assets, but the new holders of these assets and the holders of the other 3 billion in assets have a moral obligation to use them to support the people of Afghanistan.

We fervently believe that all of these funds belong to the people of Afghanistan and should be used to prevent further development of a humanitarian crisis and economic collapse. There cannot be a better time for countries and organizations to fulfill their moral responsibility and contribute to rebuilding Afghanistan, after their decades of involvement in war and conflict. In this commentary we discuss ways in which the Taliban could be engaged, and short term and long term priorities to help stabilize Afghanistan. We also describe how this problem could be prevented in the future to ensure that a sovereign nation’s assets are used to support the health and wellbeing of their people.

## Engaging the Taliban and international oversight

At this critical juncture, providing aid to the people of Afghanistan and stabilizing the economy is impossible without diplomatic relations being made with Taliban. While doing so is a much larger topic than this article, we must emphasize that the Taliban’s actions and use of funds should be closely monitored by international governing bodies. In particular, a top priority is to work with the Taliban government to ensure that the Central Bank of Afghanistan is independent, monetary flow is internationally monitored, and that funds can be frozen at any time if there is suspicion of abuse. The freezing and withholding of assets should be clearly delineated by any misuse of the Taliban. International organizations that have worked in Afghanistan in the past, such as the UN, World Bank, and International Monetary Fund (IMF) could coordinate together to fulfill this regulatory role. As the IMF has the technical skills and infrastructure to monitor financial transactions, their monitoring could be followed by enforcement from the UN or World Bank. While it will be challenging to monitor and ensure proper spending, it is essential to ensure proper disbursal of humanitarian funds and a a stable economic future for Afghanistan.

## Short term interventions

There are a number of short-term concerns in the developing humanitarian crises in Afghanistan, but widespread starvation and economic collapse remain among the highest.

It is estimated that 55% of all 43 million Afghans will face acute food insecurity in the next few months with nearly 9 million people almost living in famine conditions [3]. This issue is likely only to worsen as food storages are depleted and the drought persists. While there are a number of problems associated with the economic impacts and sustainability of direct food aid, the crisis in Afghanistan demands action now to save lives. Further support should be given to the WFP, which has been working on the ground to secure nutrition for 24 million Afghans [4]. Food aid should be diverse and also meet the nutritional needs of the 13 million children facing mal-nutrition.

As previously mentioned, an independent functioning central bank is essential to keeping the economy moving and regulated. In the short term, portions of this funding should be used as direct cash transfers, which have been shown to have positive effects on improving the economy and health outcomes in humanitarian contexts [5]. Organizations such as the WFP have already been scaling to provide cash transfers to over 6 million Afghans. However, as more organizations begin providing cash transfers, there should be further collaboration within an economic recovery cluster with input from the UN and World Bank. Putting money into the hands of Afghanis at this time is ensuring spending and economic activity. Moreover, local Afghan organizations such as Afghan Health and Development Services (AHDS), and Just For Afghan Capacity and Knowledge (JACK) should be supported to continue their work in providing health services to the population.

## Long term

In the long term, the billions in assets should be used to help grow and stabilize the country of Afghanistan. In particular the economy, healthcare, and the rights of women and children should be prioritized [6, 7].

As Afghanistan relied upon these international groups for decades, these groups should restart their work to help the country gain more financial independence. In addition to loans through an independent central bank, assets should be given to microloan organizations such as the Afghanistan Rural Microcredit Programme (ARMP) to help distribute funds quicker to the people of Afghanistan. As the Afghan people have the right to start their own businesses by investing in the private sector, it is essential that Afghan currency does not lose its value in the market.

After withdrawal of international support, Afghanistan’s largest health services provider, Sehatmandi, lost its functionality [8]. This crippled the healthcare infrastructure and healthcare personnel organizations, which need to be rebuilt [9]. Assets should be used by the UN and NGOs to first ensure that healthcare facilities are properly supplied and healthcare workers are paid. After the healthcare system is returned to a functional state, assets could be used to address the most pressing healthcare problems in the country including maternal mortality, child mental health, and malnutrition, and respiratory diseases [4, 10, 11].

As women and children are vulnerable groups in Afghanistan and essential to future prosperity of the country, a special effort should be made to ensure their success. Frozen assets should be used to leverage protections and rights for women. The assets should be used to ensure that females are allowed to pursue education and work in careers of their choosing. Funds should be used to support local NGOs already doing this work, such the Afghan Women’s Network and the Afghan Women’s Council. Female healthcare workers should be guaranteed safety to continue their work under this new regime. Given the rates of malnutrition and the mental health impact on children from the decades of conflict, child health should be made an urgent priority. Child health issues could be addressed by strengthening the education system and building support systems within education. In the education system, girls should be allowed to pursue their education in universities without any barriers. In particular, separating males and females in the education system, which creates unequal learning environments, will worsen conditions. While cultural contexts matter, it should not be a challenge for young girls and women to pursue education. As the future of Afghanistan relies on the country’s women and children these assets should be leveraged to ensure their rights and access to education.

## Conclusion

Afghanistan’s twenty-year dependance on international funding and support has now left the country without its own sustainable structures to ensure economic and physical health. The globalization of politics and economics has left the people of Afghanistan to suffer in a dire humanitarian crisis that is continuously compounded by events, including the recent earthquake. The current problem of what to do with the 10 billion in Afghan assets again clearly demonstrate the problems of globalized politics and economies in responding to health crises around the world. The death and suffering of the Afghan people call for swift and purposeful action using the systems that could have prevented such a crisis. At this critical juncture the Biden Administration, which divided the 7 billion of US held assets, but still controls their disbursement is morally and economically obligated to ensure all assets go to helping the people of Afghanistan in a transparent manner. The organization’s holding the other 3 billion in assets including the World Bank must also follow suit. Withholding these assets sets a dangerous precedent of neo-colonial international governance that allows foreign governments to keep assets belonging to a people, especially when they are in dire need of help. International law should begin to reflect the impact that globalization has on a country’s economic assets and how this connects to a people’s health and wellbeing. As the crisis in Afghanistan goes beyond deciding these assets, the US and the international allies must feel their obligation beyond what has currently plagued the country by ensuring healthcare availability, safety and prosperity of Afghanistan and its people. If action is not taken, millions of Afghans will suffer, die, and the country will face a bleak future.

The events currently unfolding in Afghanistan are a warning sign for the impact globalized politics and economics can have on a country’s health. Especially when working with hostile governments such as the Taliban, international organizations should endeavor to create independent systems for resources distribution and quality assurance. The two key lessons from Afghanistan are that international intervention in a sovereign nation necessitates an international response to reconstruct that nation and that in a globalized economy the people of a sovereign nation deserve to benefit from their countries assets even if they are held internationally.

## Data Availability

Not applicable.

## Abbreviations

WFP: World Food Program
UN: United Nation
IMF: International Monetary Fund
US: United States
